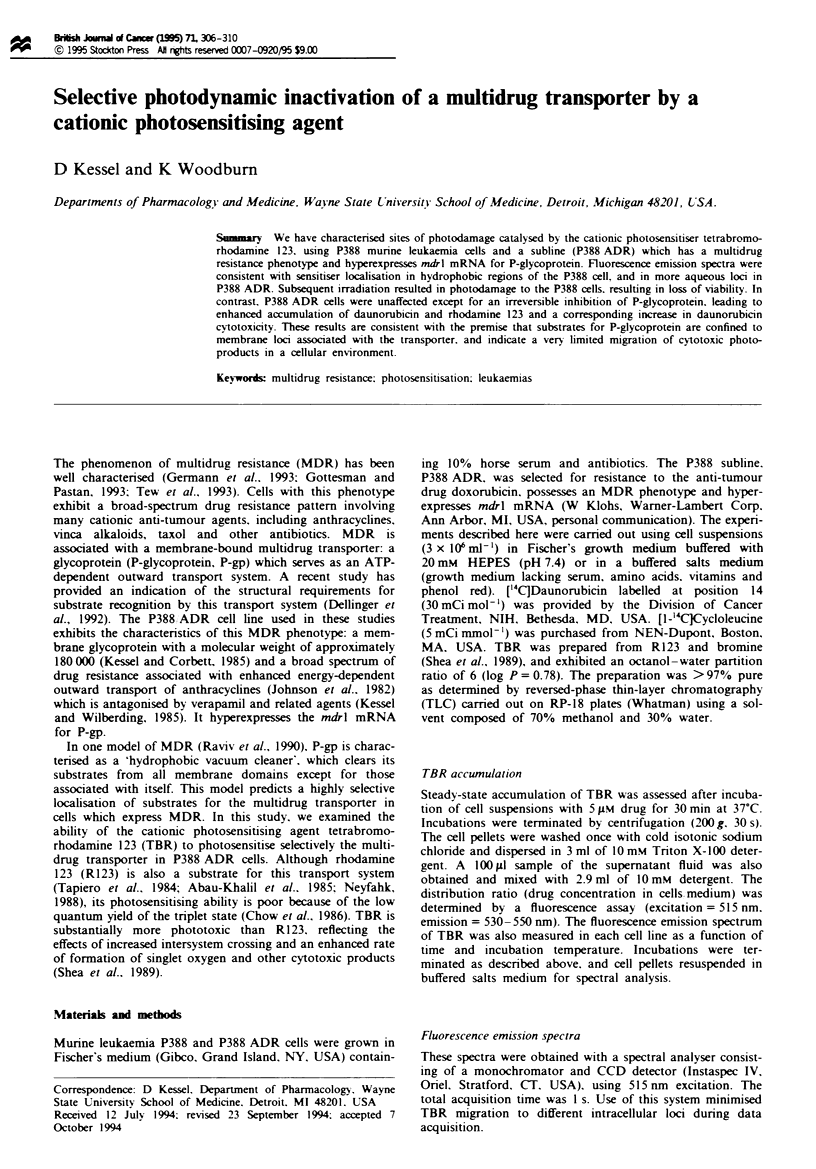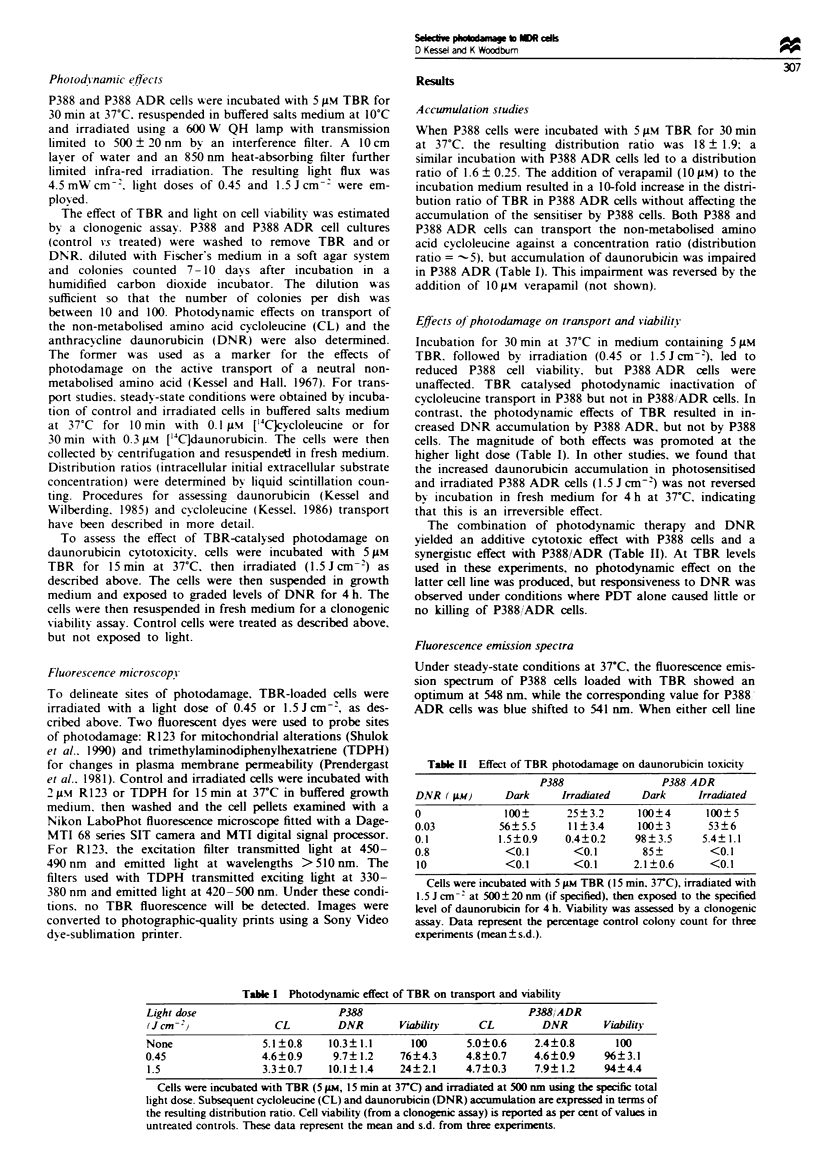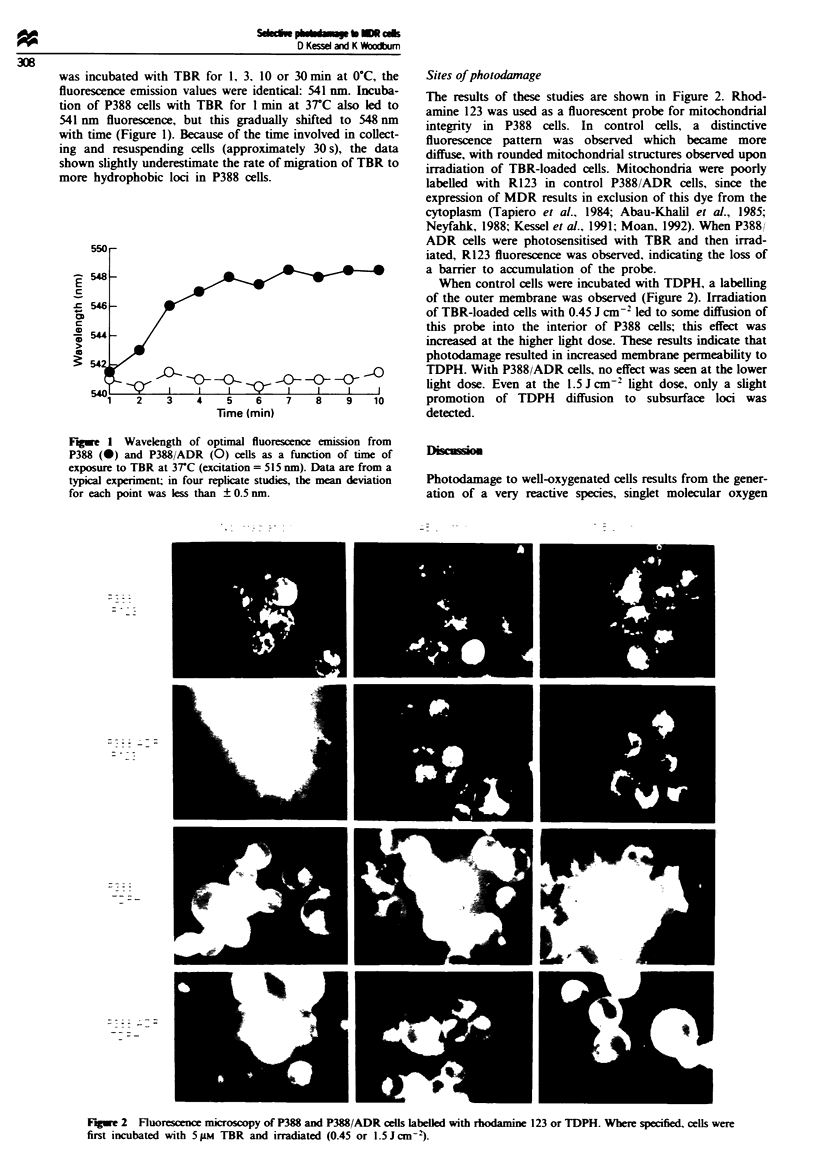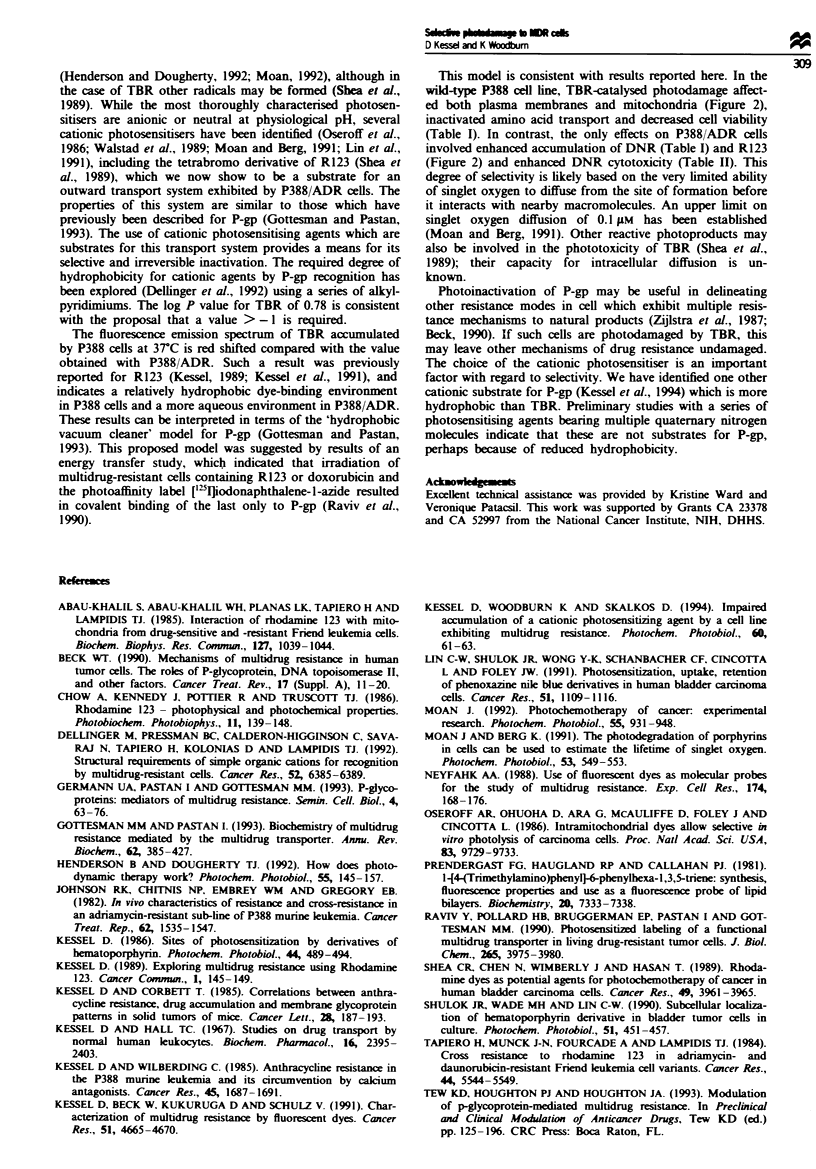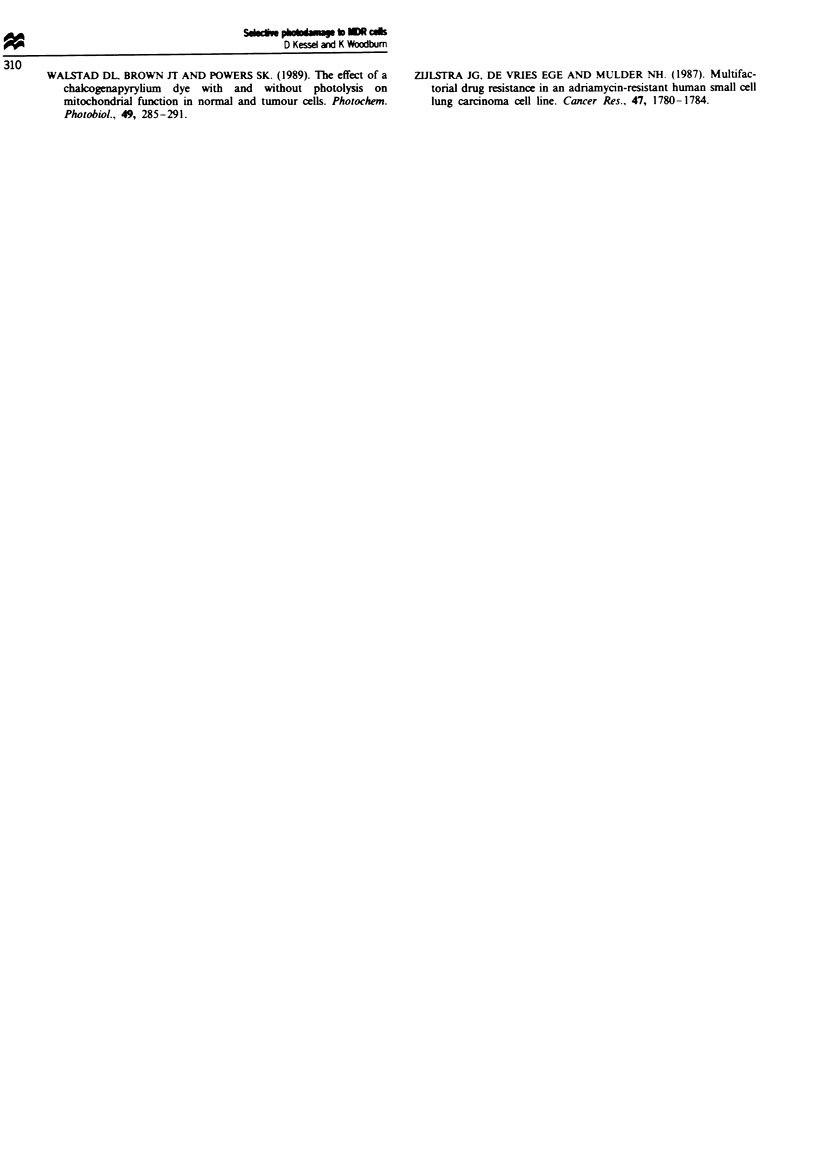# Selective photodynamic inactivation of a multidrug transporter by a cationic photosensitising agent.

**DOI:** 10.1038/bjc.1995.61

**Published:** 1995-02

**Authors:** D. Kessel, K. Woodburn

**Affiliations:** Department of Pharmacology, Wayne State University School of Medicine, Detroit, Michigan 48201.

## Abstract

**Images:**


British Journal of Cancer (1995) 71, 306-310

© 1995 Stockton Press All rights reserved 0007-0920/95 $9.00

### Selective photodynamic inactivation of a multidrug transporter by a cationic photosensitising agent

D Kessel and K Woodburn

*Departments of Pharmacology and Medicine, Wayne State University School of Medicine, Detroit, Michigan 48201, USA.*

**Summary** We have characterised sites of photodamage catalysed by the cationic photosensitiser tetrabromorhodamine 123, using P388 murine leukaemia cells and a subline (P388 ADR) which has a multidrug resistance phenotype and hyperexpresses *mdr*1 mRNA for P-glycoprotein. Fluorescence emission spectra were consistent with sensitiser localisation in hydrophobic regions of the P388 cell, and in more aqueous loci in P388 ADR. Subsequent irradiation resulted in photodamage to the P388 cells, resulting in loss of viability. In contrast, P388 ADR cells were unaffected except for an irreversible inhibition of P-glycoprotein, leading to enhanced accumulation of daunorubicin and rhodamine 123 and a corresponding increase in daunorubicin cytotoxicity. These results are consistent with the premise that substrates for P-glycoprotein are confined to membrane loci associated with the transporter, and indicate a very limited migration of cytotoxic photoproducts in a cellular environment.

**Keywords:** multidrug resistance; photosensitisation; leukaemias

The phenomenon of multidrug resistance (MDR) has been well characterised (Germann *et al.*, 1993; Gottesman and Pastan, 1993; Tew *et al.*, 1993). Cells with this phenotype exhibit a broad-spectrum drug resistance pattern involving many cationic anti-tumour agents, including anthracyclines, vinca alkaloids, taxol and other antibiotics. MDR is associated with a membrane-bound multidrug transporter: a glycoprotein (P-glycoprotein, P-gp) which serves as an ATP-dependent outward transport system. A recent study has provided an indication of the structural requirements for substrate recognition by this transport system (Dellinger *et al.*, 1992). The P388/ADR cell line used in these studies exhibits the characteristics of this MDR phenotype: a membrane glycoprotein with a molecular weight of approximately 180 000 (Kessel and Corbett, 1985) and a broad spectrum of drug resistance associated with enhanced energy-dependent outward transport of anthracyclines (Johnson *et al.*, 1982) which is antagonised by verapamil and related agents (Kessel and Wilberding, 1985). It hyperexpresses the *mdr*1 mRNA for P-gp.

In one model of MDR (Raviv *et al.*, 1990), P-gp is characterised as a 'hydrophobic vacuum cleaner', which clears its substrates from all membrane domains except for those associated with itself. This model predicts a highly selective localisation of substrates for the multidrug transporter in cells which express MDR. In this study, we examined the ability of the cationic photosensitising agent tetrabromorhodamine 123 (TBR) to photosensitise selectively the multidrug transporter in P388 ADR cells. Although rhodamine 123 (R123) is also a substrate for this transport system (Tapiero *et al.*, 1984; Abau-Khalil *et al.*, 1985; Neyfahk, 1988), its photosensitising ability is poor because of the low quantum yield of the triplet state (Chow *et al.*, 1986). TBR is substantially more phototoxic than R123, reflecting the effects of increased intersystem crossing and an enhanced rate of formation of singlet oxygen and other cytotoxic products (Shea *et al.*, 1989).

### Materials and methods

Murine leukaemia P388 and P388 ADR cells were grown in Fischer's medium (Gibco, Grand Island, NY, USA) containing 10% horse serum and antibiotics. The P388 subline, P388 ADR, was selected for resistance to the anti-tumour drug doxorubicin, possesses an MDR phenotype and hyperexpresses *mdr*1 mRNA (W Klohs, Warner-Lambert Corp, Ann Arbor, MI, USA, personal communication). The experiments described here were carried out using cell suspensions ($3 \times 10^6$ ml$^{-1}$) in Fischer's growth medium buffered with 20 mM HEPES (pH 7.4) or in a buffered salts medium (growth medium lacking serum, amino acids, vitamins and phenol red). [$^{14}$C]Daunorubicin labelled at position 14 (30 mCi mol$^{-1}$) was provided by the Division of Cancer Treatment, NIH, Bethesda, MD, USA. [1-$^{14}$C]Cycloleucine (5 mCi mmol$^{-1}$) was purchased from NEN-Dupont, Boston, MA, USA. TBR was prepared from R123 and bromine (Shea *et al.*, 1989), and exhibited an octanol-water partition ratio of 6 (log $P = 0.78$). The preparation was >97% pure as determined by reversed-phase thin-layer chromatography (TLC) carried out on RP-18 plates (Whatman) using a solvent composed of 70% methanol and 30% water.

### *TBR accumulation*

Steady-state accumulation of TBR was assessed after incubation of cell suspensions with 5 μM drug for 30 min at 37°C. Incubations were terminated by centrifugation (200 ***g***, 30 s). The cell pellets were washed once with cold isotonic sodium chloride and dispersed in 3 ml of 10 mM Triton X-100 detergent. A 100 μl sample of the supernatant fluid was also obtained and mixed with 2.9 ml of 10 mM detergent. The distribution ratio (drug concentration in cells/medium) was determined by a fluorescence assay (excitation = 515 nm, emission = 530-550 nm). The fluorescence emission spectrum of TBR was also measured in each cell line as a function of time and incubation temperature. Incubations were terminated as described above, and cell pellets resuspended in buffered salts medium for spectral analysis.

### *Fluorescence emission spectra*

These spectra were obtained with a spectral analyser consisting of a monochromator and CCD detector (Instaspec IV, Oriel, Stratford, CT, USA), using 515 nm excitation. The total acquisition time was 1 s. Use of this system minimised TBR migration to different intracellular loci during data acquisition.

Correspondence: D Kessel, Department of Pharmacology, Wayne State University School of Medicine, Detroit, MI 48201, USA
Received 12 July 1994; revised 23 September 1994; accepted 7 October 1994

### Photodynamic effects

P388 and P388 ADR cells were incubated with 5 μM TBR for 30 min at 37°C, resuspended in buffered salts medium at 10°C and irradiated using a 600 W QH lamp with transmission limited to 500 ± 20 nm by an interference filter. A 10 cm layer of water and an 850 nm heat-absorbing filter further limited infra-red irradiation. The resulting light flux was 4.5 mW $cm^{-2}$, light doses of 0.45 and 1.5 J $cm^{-2}$ were employed.

The effect of TBR and light on cell viability was estimated by a clonogenic assay. P388 and P388 ADR cell cultures (control *vs* treated) were washed to remove TBR and or DNR, diluted with Fischer's medium in a soft agar system and colonies counted 7–10 days after incubation in a humidified carbon dioxide incubator. The dilution was sufficient so that the number of colonies per dish was between 10 and 100. Photodynamic effects on transport of the non-metabolised amino acid cycloleucine (CL) and the anthracycline daunorubicin (DNR) were also determined. The former was used as a marker for the effects of photodamage on the active transport of a neutral non-metabolised amino acid (Kessel and Hall, 1967). For transport studies, steady-state conditions were obtained by incubation of control and irradiated cells in buffered salts medium at 37°C for 10 min with 0.1 μM [$^{14}$C]cycloleucine or for 30 min with 0.3 μM [$^{14}$C]daunorubicin. The cells were then collected by centrifugation and resuspended in fresh medium. Distribution ratios (intracellular initial extracellular substrate concentration) were determined by liquid scintillation counting. Procedures for assessing daunorubicin (Kessel and Wilberding, 1985) and cycloleucine (Kessel, 1986) transport have been described in more detail.

To assess the effect of TBR-catalysed photodamage on daunorubicin cytotoxicity, cells were incubated with 5 μM TBR for 15 min at 37°C, then irradiated (1.5 J $cm^{-2}$) as described above. The cells were then suspended in growth medium and exposed to graded levels of DNR for 4 h. The cells were then resuspended in fresh medium for a clonogenic viability assay. Control cells were treated as described above, but not exposed to light.

### Fluorescence microscopy

To delineate sites of photodamage, TBR-loaded cells were irradiated with a light dose of 0.45 or 1.5 J $cm^{-2}$, as described above. Two fluorescent dyes were used to probe sites of photodamage: R123 for mitochondrial alterations (Shulok *et al.*, 1990) and trimethylaminodiphenylhexatriene (TDPH) for changes in plasma membrane permeability (Prendergast *et al.*, 1981). Control and irradiated cells were incubated with 2 μM R123 or TDPH for 15 min at 37°C in buffered growth medium, then washed and the cell pellets examined with a Nikon LaboPhot fluorescence microscope fitted with a Dage-MTI 68 series SIT camera and MTI digital signal processor. For R123, the excitation filter transmitted light at 450–490 nm and emitted light at wavelengths >510 nm. The filters used with TDPH transmitted exciting light at 330–380 nm and emitted light at 420–500 nm. Under these conditions, no TBR fluorescence will be detected. Images were converted to photographic-quality prints using a Sony Video dye-sublimation printer.

### Results

### Accumulation studies

When P388 cells were incubated with 5 μM TBR for 30 min at 37°C, the resulting distribution ratio was 18 ± 1.9; a similar incubation with P388 ADR cells led to a distribution ratio of 1.6 ± 0.25. The addition of verapamil (10 μM) to the incubation medium resulted in a 10-fold increase in the distribution ratio of TBR in P388 ADR cells without affecting the accumulation of the sensitiser by P388 cells. Both P388 and P388 ADR cells can transport the non-metabolised amino acid cycloleucine against a concentration ratio (distribution ratio = ~5), but accumulation of daunorubicin was impaired in P388 ADR (Table I). This impairment was reversed by the addition of 10 μM verapamil (not shown).

### Effects of photodamage on transport and viability

Incubation for 30 min at 37°C in medium containing 5 μM TBR, followed by irradiation (0.45 or 1.5 J $cm^{-2}$), led to reduced P388 cell viability, but P388 ADR cells were unaffected. TBR catalysed photodynamic inactivation of cycloleucine transport in P388 but not in P388/ADR cells. In contrast, the photodynamic effects of TBR resulted in increased DNR accumulation by P388 ADR, but not by P388 cells. The magnitude of both effects was promoted at the higher light dose (Table I). In other studies, we found that the increased daunorubicin accumulation in photosensitised and irradiated P388 ADR cells (1.5 J $cm^{-2}$) was not reversed by incubation in fresh medium for 4 h at 37°C, indicating that this is an irreversible effect.

The combination of photodynamic therapy and DNR yielded an additive cytotoxic effect with P388 cells and a synergistic effect with P388/ADR (Table II). At TBR levels used in these experiments, no photodynamic effect on the latter cell line was produced, but responsiveness to DNR was observed under conditions where PDT alone caused little or no killing of P388/ADR cells.

### Fluorescence emission spectra

Under steady-state conditions at 37°C, the fluorescence emission spectrum of P388 cells loaded with TBR showed an optimum at 548 nm, while the corresponding value for P388/ADR cells was blue shifted to 541 nm. When either cell line

**Table II** Effect of TBR photodamage on daunorubicin toxicity

| | *P388* | | *P388/ADR* | |
|---|---|---|---|---|
| *DNR (μM)* | *Dark* | *Irradiated* | *Dark* | *Irradiated* |
| 0 | 100± | 25±3.2 | 100±4 | 100±5 |
| 0.03 | 56±5.5 | 11±3.4 | 100±3 | 53±6 |
| 0.1 | 1.5±0.9 | 0.4±0.2 | 98±3.5 | 5.4±1.1 |
| 0.8 | <0.1 | <0.1 | 85± | <0.1 |
| 10 | <0.1 | <0.1 | 2.1±0.6 | <0.1 |

Cells were incubated with 5 μM TBR (15 min, 37°C), irradiated with 1.5 J $cm^{-2}$ at 500±20 nm (if specified), then exposed to the specified level of daunorubicin for 4 h. Viability was assessed by a clonogenic assay. Data represent the percentage control colony count for three experiments (mean±s.d.).

**Table I** Photodynamic effect of TBR on transport and viability

| *Light dose* | | *P388* | | | *P388/ADR* | |
|---|---|---|---|---|---|---|
| *(J $cm^{-2}$)* | *CL* | *DNR* | *Viability* | *CL* | *DNR* | *Viability* |
| None | 5.1±0.8 | 10.3±1.1 | 100 | 5.0±0.6 | 2.4±0.8 | 100 |
| 0.45 | 4.6±0.9 | 9.7±1.2 | 76±4.3 | 4.8±0.7 | 4.6±0.9 | 96±3.1 |
| 1.5 | 3.3±0.7 | 10.1±1.4 | 24±2.1 | 4.7±0.3 | 7.9±1.2 | 94±4.4 |

Cells were incubated with TBR (5 μM, 15 min at 37°C) and irradiated at 500 nm using the specific total light dose. Subsequent cycloleucine (CL) and daunorubicin (DNR) accumulation are expressed in terms of the resulting distribution ratio. Cell viability (from a clonogenic assay) is reported as per cent of values in untreated controls. These data represent the mean and s.d. from three experiments.

was incubated with TBR for 1, 3, 10 or 30 min at 0°C, the fluorescence emission values were identical: 541 nm. Incubation of P388 cells with TBR for 1 min at 37°C also led to 541 nm fluorescence, but this gradually shifted to 548 nm with time (Figure 1). Because of the time involved in collecting and resuspending cells (approximately 30 s), the data shown slightly underestimate the rate of migration of TBR to more hydrophobic loci in P388 cells.

**Figure 1** Wavelength of optimal fluorescence emission from P388 (●) and P388 ADR (○) cells as a function of time of exposure to TBR at 37°C (excitation = 515 nm). Data are from a typical experiment; in four replicate studies, the mean deviation for each point was less than ± 0.5 nm.

*Sites of photodamage*

The results of these studies are shown in Figure 2. Rhodamine 123 was used as a fluorescent probe for mitochondrial integrity in P388 cells. In control cells, a distinctive fluorescence pattern was observed which became more diffuse, with rounded mitochondrial structures observed upon irradiation of TBR-loaded cells. Mitochondria were poorly labelled with R123 in control P388 ADR cells, since the expression of MDR results in exclusion of this dye from the cytoplasm (Tapiero *et al.*, 1984; Abau-Khalil *et al.*, 1985; Neyfahk, 1988; Kessel *et al.*, 1991; Moan, 1992). When P388 ADR cells were photosensitised with TBR and then irradiated, R123 fluorescence was observed, indicating the loss of a barrier to accumulation of the probe.

When control cells were incubated with TDPH, a labelling of the outer membrane was observed (Figure 2). Irradiation of TBR-loaded cells with 0.45 J $cm^{-2}$ led to some diffusion of this probe into the interior of P388 cells; this effect was increased at the higher light dose. These results indicate that photodamage resulted in increased membrane permeability to TDPH. With P388 ADR cells, no effect was seen at the lower light dose. Even at the 1.5 J $cm^{-2}$ light dose, only a slight promotion of TDPH diffusion to subsurface loci was detected.

### Discussion

Photodamage to well-oxygenated cells results from the generation of a very reactive species, singlet molecular oxygen

**Figure 2** Fluorescence microscopy of P388 and P388 ADR cells labelled with rhodamine 123 or TDPH. Where specified, cells were first incubated with 5 μM TBR and irradiated (0.45 or 1.5 J $cm^{-2}$).

(Henderson and Dougherty, 1992; Moan, 1992), although in the case of TBR other radicals may be formed (Shea *et al.*, 1989). While the most thoroughly characterised photosensitisers are anionic or neutral at physiological pH, several cationic photosensitisers have been identified (Oseroff *et al.*, 1986; Walstad *et al.*, 1989; Moan and Berg, 1991; Lin *et al.*, 1991), including the tetrabromo derivative of R123 (Shea *et al.*, 1989), which we now show to be a substrate for an outward transport system exhibited by P388 ADR cells. The properties of this system are similar to those which have previously been described for P-gp (Gottesman and Pastan, 1993). The use of cationic photosensitising agents which are substrates for this transport system provides a means for its selective and irreversible inactivation. The required degree of hydrophobicity for cationic agents by P-gp recognition has been explored (Dellinger *et al.*, 1992) using a series of alkylpyridimiums. The log *P* value for TBR of 0.78 is consistent with the proposal that a value $> -1$ is required.

The fluorescence emission spectrum of TBR accumulated by P388 cells at 37°C is red shifted compared with the value obtained with P388 ADR. Such a result was previously reported for R123 (Kessel, 1989; Kessel *et al.*, 1991), and indicates a relatively hydrophobic dye-binding environment in P388 cells and a more aqueous environment in P388 ADR. These results can be interpreted in terms of the 'hydrophobic vacuum cleaner' model for P-gp (Gottesman and Pastan, 1993). This proposed model was suggested by results of an energy transfer study, which indicated that irradiation of multidrug-resistant cells containing R123 or doxorubicin and the photoaffinity label [$^{125}$I]iodonaphthalene-1-azide resulted in covalent binding of the last only to P-gp (Raviv *et al.*, 1990).

This model is consistent with results reported here. In the wild-type P388 cell line, TBR-catalysed photodamage affected both plasma membranes and mitochondria (Figure 2), inactivated amino acid transport and decreased cell viability (Table I). In contrast, the only effects on P388 ADR cells involved enhanced accumulation of DNR (Table I) and R123 (Figure 2) and enhanced DNR cytotoxicity (Table II). This degree of selectivity is likely based on the very limited ability of singlet oxygen to diffuse from the site of formation before it interacts with nearby macromolecules. An upper limit on singlet oxygen diffusion of 0.1 μM has been established (Moan and Berg, 1991). Other reactive photoproducts may also be involved in the phototoxicity of TBR (Shea *et al.*, 1989); their capacity for intracellular diffusion is unknown.

Photoinactivation of P-gp may be useful in delineating other resistance modes in cell which exhibit multiple resistance mechanisms to natural products (Zijlstra *et al.*, 1987; Beck, 1990). If such cells are photodamaged by TBR, this may leave other mechanisms of drug resistance undamaged. The choice of the cationic photosensitiser is an important factor with regard to selectivity. We have identified one other cationic substrate for P-gp (Kessel *et al.*, 1994) which is more hydrophobic than TBR. Preliminary studies with a series of photosensitising agents bearing multiple quaternary nitrogen molecules indicate that these are not substrates for P-gp, perhaps because of reduced hydrophobicity.

**Acknowledgements**
Excellent technical assistance was provided by Kristine Ward and Veronique Patacsil. This work was supported by Grants CA 23378 and CA 52997 from the National Cancer Institute, NIH, DHHS.

### References

ABAU-KHALIL S, ABAU-KHALIL WH, PLANAS LK, TAPIERO H AND LAMPIDIS TJ. (1985). Interaction of rhodamine 123 with mitochondria from drug-sensitive and -resistant Friend leukemia cells. *Biochem. Biophys. Res. Commun.*, **127**, 1039–1044.

BECK WT. (1990). Mechanisms of multidrug resistance in human tumor cells. The roles of P-glycoprotein, DNA topoisomerase II, and other factors. *Cancer Treat. Rev.*, **17** (Suppl. A), 11–20.

CHOW A, KENNEDY J, POTTIER R AND TRUSCOTT TJ. (1986). Rhodamine 123 – photophysical and photochemical properties. *Photobiochem. Photobiophys.*, **11**, 139–148.

DELLINGER M, PRESSMAN BC, CALDERON-HIGGINSON C, SAVARAJ N, TAPIERO H, KOLONIAS D AND LAMPIDIS TJ. (1992). Structural requirements of simple organic cations for recognition by multidrug-resistant cells. *Cancer Res.*, **52**, 6385–6389.

GERMANN UA, PASTAN I AND GOTTESMAN MM. (1993). P-glycoproteins: mediators of multidrug resistance. *Semin. Cell. Biol.*, **4**, 63–76.

GOTTESMAN MM AND PASTAN I. (1993). Biochemistry of multidrug resistance mediated by the multidrug transporter. *Annu. Rev. Biochem.*, **62**, 385–427.

HENDERSON B AND DOUGHERTY TJ. (1992). How does photodynamic therapy work? *Photochem. Photobiol.*, **55**, 145–157.

JOHNSON RK, CHITNIS NP, EMBREY WM AND GREGORY EB. (1982). *In vivo* characteristics of resistance and cross-resistance in an adriamycin-resistant sub-line of P388 murine leukemia. *Cancer Treat. Rep.*, **62**, 1535–1547.

KESSEL D. (1986). Sites of photosensitization by derivatives of hematoporphyrin. *Photochem. Photobiol.*, **44**, 489–494.

KESSEL D. (1989). Exploring multidrug resistance using Rhodamine 123. *Cancer Commun.*, **1**, 145–149.

KESSEL D AND CORBETT T. (1985). Correlations between anthracycline resistance, drug accumulation and membrane glycoprotein patterns in solid tumors of mice. *Cancer Lett.*, **28**, 187–193.

KESSEL D AND HALL TC. (1967). Studies on drug transport by normal human leukocytes. *Biochem. Pharmacol.*, **16**, 2395–2403.

KESSEL D AND WILBERDING C. (1985). Anthracycline resistance in the P388 murine leukemia and its circumvention by calcium antagonists. *Cancer Res.*, **45**, 1687–1691.

KESSEL D, BECK W, KUKURUGA D AND SCHULZ V. (1991). Characterization of multidrug resistance by fluorescent dyes. *Cancer Res.*, **51**, 4665–4670.

KESSEL D, WOODBURN K AND SKALKOS D. (1994). Impaired accumulation of a cationic photosensitizing agent by a cell line exhibiting multidrug resistance. *Photochem. Photobiol.*, **60**, 61–63.

LIN C-W, SHULOK JR, WONG Y-K, SCHANBACHER CF, CINCOTTA L AND FOLEY JW. (1991). Photosensitization, uptake, retention of phenoxazine nile blue derivatives in human bladder carcinoma cells. *Cancer Res.*, **51**, 1109–1116.

MOAN J. (1992). Photochemotherapy of cancer: experimental research. *Photochem. Photobiol.*, **55**, 931–948.

MOAN J AND BERG K. (1991). The photodegradation of porphyrins in cells can be used to estimate the lifetime of singlet oxygen. *Photochem. Photobiol.*, **53**, 549–553.

NEYFAHK AA. (1988). Use of fluorescent dyes as molecular probes for the study of multidrug resistance. *Exp. Cell Res.*, **174**, 168–176.

OSEROFF AR, OHUOHA D, ARA G, MCAULIFFE D, FOLEY J AND CINCOTTA L. (1986). Intramitochondrial dyes allow selective *in vitro* photolysis of carcinoma cells. *Proc. Natl Acad. Sci. USA*, **83**, 9729–9733.

PRENDERGAST FG, HAUGLAND RP AND CALLAHAN PJ. (1981). 1-[4-(Trimethylamino)phenyl]-6-phenylhexa-1,3,5-triene: synthesis, fluorescence properties and use as a fluorescence probe of lipid bilayers. *Biochemistry*, **20**, 7333–7338.

RAVIV Y, POLLARD HB, BRUGGERMAN EP, PASTAN I AND GOTTESMAN MM. (1990). Photosensitized labeling of a functional multidrug transporter in living drug-resistant tumor cells. *J. Biol. Chem.*, **265**, 3975–3980.

SHEA CR, CHEN N, WIMBERLY J AND HASAN T. (1989). Rhodamine dyes as potential agents for photochemotherapy of cancer in human bladder carcinoma cells. *Cancer Res.*, **49**, 3961–3965.

SHULOK JR, WADE MH AND LIN C-W. (1990). Subcellular localization of hematoporphyrin derivative in bladder tumor cells in culture. *Photochem. Photobiol.*, **51**, 451–457.

TAPIERO H, MUNCK J-N, FOURCADE A AND LAMPIDIS TJ. (1984). Cross resistance to rhodamine 123 in adriamycin- and daunorubicin-resistant Friend leukemia cell variants. *Cancer Res.*, **44**, 5544–5549.

TEW KD, HOUGHTON PJ AND HOUGHTON JA. (1993). Modulation of p-glycoprotein-mediated multidrug resistance. In *Preclinical and Clinical Modulation of Anticancer Drugs*, Tew KD (ed.) pp. 125–196. CRC Press: Boca Raton, FL.

WALSTAD DL, BROWN JT AND POWERS SK. (1989). The effect of a chalcogenapyrylium dye with and without photolysis on mitochondrial function in normal and tumour cells. *Photochem. Photobiol.*, **49**, 285–291.

ZIJLSTRA JG, DE VRIES EGE AND MULDER NH. (1987). Multifactorial drug resistance in an adriamycin-resistant human small cell lung carcinoma cell line. *Cancer Res.*, **47**, 1780–1784.